# The Evaluation of Fibrin Sealants and Tissue Adhesives in Oral Surgery Among Patients with Bleeding Disorders

**DOI:** 10.5505/tjh.2012.07769

**Published:** 2012-03-05

**Authors:** Gülsüm Ak, Esra Alpkılıç Başkırt, Esma Kürklü, Meltem Koray, Hakkı Tanyeri, Bülent Zülfikar

**Affiliations:** 1 Istanbul University, Faculty of Dentistry, Department of Oral Surgery and Medicine , İstanbul, Turkey; 2 Hospitadent Oral and Dental Health Rehabilitation Center, Oral Surgery Department, İstanbul, Turkey; 3 İstanbul University, Cerrahpaşa School of Medicine, Department of Pediatric Hematology-Oncology, İstanbul, Turkey

**Keywords:** Fibrin sealants, Tissue adhesive, Tooth extraction, Hemophilia, Bleeding disorders

## Abstract

**Objective:** The aim of this study was to evaluate the efficiency of two local hemostatic agents administered with apreoperative dose of replacement therapy in patients with bleeding disorders undergoing oral surgery.

**Material and Methods:** The study included 21 patients that were randomly divided into 3 groups. Patients in Group1 (n = 7) received preoperative replacement therapy and postoperative fibrin sealant applied to the surgical site. Patientsin Group 2 (n = 7) received preoperative replacement therapy and postoperative tissue adhesive applied to the surgicalsite. Patients in Group 3 (n = 7) were given replacement therapy pre- and postoperatively.

**Results:** Postoperative bleeding was not observed in 17 of the 21 patients, including 5 in Group 1 (71.42%), 6 in Group2 (85.71%), and 6 in Group 3 (85.71%). Hemorrhagic complications occurred in only 4 of the 21 patients.

**Conclusion:** The use of fibrin sealant and tissue adhesive was beneficial, as they reduced the level of factor concentratesused for replacement therapy and resulted in rapid hemostasis at the surgical site, facilitating the ability to performserial surgical procedures concurrently.

## Abstract (tr)

**Amaç:** Bu çalışmanın amacı kanama bozukluğu olan hastalarda, oral cerrahi girişimlerde replasman tedavisinin yalnızca preoperatif dozu uygulanarak cerrahi alana lokal doku yapıştırıcı veya fibrin sealant uygulamasının hemostaz üzerindeki etkinliğini değerlendirmektir.

**Gereç ve Yöntemler:** Kanama bozukluğu olan 21 hasta randomize olarak üç farklı gruba ayrılmıştır. Grup 1’deki hastalara (n=7) preoperatif replasman tedavisi ile fibrin sealant uygulanmıştır. Grup 2’deki hastalara (n=7) preoperatif replasman tedavisi ile doku yapıştırıcısı uygulanmıştır. Grup 3’deki hastalara preoperatif ve postoperatif replasman tedavisi uygulanmıştır.

**Bulgular:** Grup 1’de beş hasta (%71,42), Grup 2’de altı hasta (%85,71) ve Grup 3’de altı hasta (%85,71) olmak üzere toplam 17 hastada hiçbir postoperatif komplikasyon gözlenmemiştir. Bütün gruplardan olmak üzere sadece dört hastada hemorajik komplikasyon gözlenmiştir.

**Sonuç:** Bu klinik çalışmanın sonuçlarına dayanarak, fibrin sealant ve doku yapıştırıcılarının oral cerrahi işlemlerde uygulanmasının replasman tedavisinde kullanılan faktör konsantresi miktarının azaltılması ve operasyon alanında hemostaz sağlanarak aynı anda çok sayıda işlemin yapılabilmesine olanak vermesi açısından faydalı olduğu görüşündeyiz.

## INTRODUCTION

Patients with bleeding disorders that require oral surgerydue to hemorrhage have a high risk of prolonged orexcessive bleeding. Multiple transfusions and prolongedhospitalization associated with oral surgery had previouslybeen a necessity for this group of patients. Subsequently,the introduction of clotting factor concentrates and antifibrinolyticagents has facilitated the use of oral surgicalprocedures in patients with bleeding disorders [1]. Thus,various unconventional methods are used in patients withbleeding disorders, particularly the application of fibrinsealants and tissue adhesives, as adjunctive treatment or insome cases primary treatment.

Fibrin sealants contain fibrinogen, factor XIII, thrombin,and aprotinin. Following application to the toothextraction site, thrombin converts fibrinogen to an unstablefibrin clot and aprotinin prevents clot degradation[2,3]. Traditionally, fibrin sealants are used as tissue adhesiveand hemostatic agent, as well as in new and creative ways, such as for cellular growth stimulation in tissueengineering [4,5].

Ethyl-2 cyanoa crylate, a rapid polymerizable liquidmonomer, is a synthetic tissue adhesive for topical use.Upon application, liquid monomer formulation polymerizesinstantly into a thin flexible polymer film that adheresstrongly to oral tissues [6,7]; however, the mechanism bywhich cyanoacrylate glue generates hemostas is is unclear.The hypothesis is that the ester forms a microfilm thatcauses mechanical blockage that slows blood flow, providinga surface agent to activate the clotting cascade[8]. There is evidence that the film forms a porous massthat is invaded by blood with subsequent clotting withinthe pores of the adhesive. As such, the rationale behind the use of ethyl-2 cyanoacrylate is to hold the opposing wound edges together while also functioning as a wounddressing that enhances clot formation or as a clot stabilizer[9]. The clinical features and benefits can be altered by adding different side chains [10]. As with fibrin sealants,tissue adhesives are widely used for oral and general surgicalinterventions, such as root canal treatment and embolotherapyfor complex cerebral and extracerebral vascularanomalies [11].

The aim of the present study was to evaluate the hemostaticefficacy of a fibrin sealant and a tissue adhesive, with only preoperative administration of replacement therapy inpatients with bleeding disorders undergoing oral surgery.

## MATERIALS AND METHODS

The study included 21 patients with bleeding disorderswho were referred for dental assessment. In all, 15patients had Hemophilia A (5 had the severe type, 7 hadthe moderate type, and 3 had the mild type), 1 patient hadHemophilia B, and 5 patients had von Willebrand disease(vWD). The male:female ratio was 3.2:1 and mean agewas 22.28 years (range: 6-40 years). Each patient underwentan initial consultation to establish a dental treatmentplan. Severe dental caries, dental abscess, and prolongedretention of deciduous teeth were the indications for toothextraction. Surgical intervention included tooth extraction,subgingival scaling, and frenectomy. Informed consentwas obtained from each patient or their parents.

**Figure 1:**
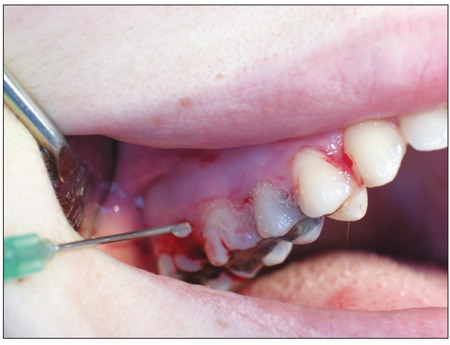
Application of fibrin sealant for gingival hemorrhagein Case 1 in Group 1.

**Figure 2:**
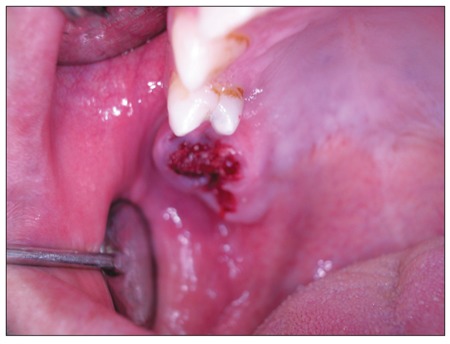
Application of tissue adhesive in the extraction socketin Case 2 in Group 2.

The patients were randomly divided into 3 groups,regardless of their disease type and severity. The replacementtherapy procedure was carried out in collaborationwith the Department of Pediatric Hematology-Oncology(Table 1). Group 1 (n=7) received preoperative replacementtherapy and postoperative fibrin sealant applied to thesurgical site.Figure 1 Group 2 (n=7) received preoperative replacementtherapy and postoperative tissue adhesive applied tothe surgical site. Postoperative replacement therapy wouldnot be given unless hemostasis was achieved in Groups 1and 2. Group 3 (n=7) received the total dose of replacementtherapy pre- and postoperatively.Figure 2 Oral surgicalprocedures were performed with minimal trauma to surroundingtissues. The distribution of patients according to sealhematologicdisease and oral surgical procedure is givenin Tables 2, 3 and 4. Subgingival scaling was performed intwo patients who had spontaneous gingival bleeding dueto mild gingivitis. Frenectomy of the inferior labial frenulumwas performed in 1 patient due to diastema betweenthe incisors.

Deciduous and permanent teeth (except for the permanentmandibular molar) were extracted after administration of articaine HCl (40 mg mL–1) and epinephrine HCl(0.006 mg mL–1) (Ultracaine DS, Aventis), either locallyor intraligamentally. Extraction of permanent mandibularmolar was performed with the inferior alveolar nerveblock. Teeth were extracted with minimal trauma to surroundingtissues. The socket was curetted and the extractioncavity was filled with 14x7x7 mm gelatin sponges(Gelatamp, Roeko, Germany) in all patients. Fibrin sealhematologic ant 0.5 mL (TisseelTM Kit, Eczacıbaşı, Baxter, Turkey) wasused for patients in Group 1 and tissue adhesive (0.3 ml/cc in tubes of 3g) (Epiglu® Meyer-Haake, Germany) wasused for patients in Group 2. Patients in Group 3 were notadministered additional local therapy. All patients weremonitored until the cessation of bleeding at the surgicalsite and were informed about postoperative care.

## RESULTS

Patient demographics, surgical procedures, and postoperativeoutcomes are summarized in Tables 2, 3 and 4.A total of 21 patients with bleeding disorders underwentoral surgery for tooth extraction (44 teeth including 12deciduous and 32 permanent), subgingival scaling (n=2),sumandfrenectomy (n=1). Postoperative hemostasis wasachieved in 17 patients, with a success rate of 71.4% (n= 5) in Group 1, 85.7% (n=6) in Group 2, and 85.7% inGroup 3 (n=6).

Hemorrhagic complications occurred in only four ofthe 21 patients. In Group 1; Case 1 (vWD) had undergonea single tooth extraction and subgingival scaling, andCase 5 (severe Hemophilia A) had one tooth extraction.To achieve hemostasis in these patients additional replacementtherapy was given postoperatively. In Group 2 Case2 (severe Hemophilia A) had 6 teeth extracted and postoperativehemorrhage occurred only at the site of needle penetration in the palatal mucosa. Tissue adhesive wasapplied once again and the bleeding ceased. In Group3 mild postoperative bleeding was observed in Case 5(severe Hemophilia A) following extraction of two teeth;complete clotting was achieved with local administrationof tranexamic acid.

## DISCUSSION

fibrin sealant and tissue adhesive, with the addition ofonly preoperative replacement therapy to control hemorrhagein patients with bleeding disorders undergoing oralsurgery. To the best of our knowledge this is the first suchstudy, and therefore the results could not be comparedto those of other studies. Hemorrhagic complicationsoccurred in only 4 of 21 patients, of which one had vWD,two had Hemophilia A, and one had Hemophilia B. It isinteresting to note that one of these patients (Case 2 fromGroup 2 [Hemophilia A]) had bleeding at the site of needlepenetration, but not in the extraction cavity. In Group 2preoperative replacement therapy and local application oftissue adhesive resulted in a 100% success rate. Additionalpostoperative replacement therapy was given to patientsin Groups 1 and 2 that had postoperative bleeding despitelocal application of fibrin sealant or tissue adhesive. Basedon the results of the present study we conclude that localapplication of fibrin sealant or tissue adhesive, togetherwith preoperative replacement therapy is a reliable andefficacious procedure in patients with bleeding disordersundergoing oral surgery. In addition, the observed reductionin blood loss during and after oral surgery facilitatesmultiple surgical procedures during a single session ofreplacement therapy, providing economical and rapidpatient rehabilitation.

Currently available treatment options for achievinghemostasis in patients with bleeding disorders are primarilyfactor replacement therapy, release of endogenous factorstores, and clot stabilization [9,12]. Factor replacementtherapy is the golden standard treatment for hemophilia;however, its high cost is a major disadvantage, and as suchrecent research has focused on local hemostatic agents,such as fibrin sealants and tissue adhesives. Satisfactoryhemorrhage control with the use of these materials has beenreported [12,13]. The primary benefits of these hemostaticagents are a life-saving reduction in hemorrhage causedby trauma, a reduction in factor dependency, a reductionin the cost of treatment, and rapid control of hemorrhage,which reduces patient anxiety related to uncontrollablebleeding [13]. Other features of these agents regardingavailability, application, safety and complication are summarized in Table 5 with a comparison to each other indicatingsuperiority.

Suturing plays an important role in controlling hemorrhage,especially in patients with bleeding disordersundergoing tooth extraction. It has been suggested thatwhen suturing is not performed (e.g. in children, as sometimessuturing is impossible due to a flat socket) saliva,due its potent fibrinolytic activity, dissolves and washesfibrin sealant away from the socket within a short time.Tongue movements also contribute to the mechanicalremoval of a clot from the socket [14]. A celluloid splint,as Suwannuraks suggested may prevent the tongue fromscratching the inside of the socket, therefore the clot wouldbe safe beneath the layer of the fibrin sealant [14]. On theother hand, cyanoacrylate functions well in a moist environment.Because moist environment does not interferewith the firm structure of the material, following applicationof tissue adhesives the enviroment need not be completelydry. Moreover, ethyl-2 cyanoacrylate—a monomerin liquid form—polymerizes in an exothermic reactionwhen in contact with a fluid medium, thereby forming abond that strongly holds together the opposing edges of awound [15]. Adhesion is achieved via attraction betweenthe molecules of an adhesives and tissue surfaces [8].These properties represent an advantage over fibrin sealants.Utilization of tissue adhesive functions as suturingdoes in providing hemostasis. Additionally, tissue adhesives with their inert feature show no resolution in saliva.These advantages were observed in the present study inGroup 2, which received Epiglue® Meyer-Haake tissueadhesive; saturation and complications were not observed.Nevertheless, application in highly moist surfaces is challengingbecause the material sets quickly when it contactswith excess liquid that is comprised of blood and/or saliva.

Fibrin sealants are derived from plasma and thereforecarry a similar risk of viral transmission as other similarplasma products; however, Kavaklı reported that duringthe last 20 years, no commercial fibrin sealant product hasbeen reported to be responsible for viral transmission, asthe result of modern viral inactivation procedures [16].Nevertheless, it is well known that a viral infection cannotbe detected during the initial incubation period of aninfectious disease—a time period during which a donor isunaware of infection and laboratory serological assays arenot able to detect the presence of a virus. As such, nonautologousfibrin sealants can carry a potential risk forviral transmission and homemade autologous fibrin sealantsappear to be a good treatment choice; however, theyare more expensive than other types of fibrin sealants. Onthe other hand, tissue adhesives are not associated withthe risk of microbial transmission, as they are synthetic,and they exhibit bacteriostatic activity on wound surfacesand are less expensive than fibrin sealant products.

Based on the limited available data, tissue adhesivesappear to have several advantages. However, their potentialfor forming exothermic heat during polymerizationand residual monomer appears as a serious disadvantage.As the polymerization chain which occurs during therecurrent or thick applications becomes longer, the risk ofexothermic heat increases.

In terms of cost-effectiveness, the treatment protocol inGroup 2 was the best, due to the lower price of tissue adhesive(as compared tot fibrin sealant) and the lower dose offactor replacement. Patients in Groups 1 and 2 receivedonly one dose of preoperative replacement therapy (inpatients with severe and moderate-mild Hemophilia A:25 U kg–1 and 20 U kg–1 of FVIII, respectively; in thosewith Hemophilia B: 40 U kg–1 of FVIX); however, patientsin Group 3 also received postoperative replacement therapyon postoperative d 1-3—a total of 55 U kg–1 for eachpatient with Hemophilia A and 110 U kg–1 for those withHemophilia B. As clotting factor concentrates are costlycomponents of hemostatic therapy in patients with bleedingdisorders, a reduction in their use will directly reducethe cost of therapy. In the UK it has been estimated thatin terms of medication alone the cost per bleeding episodein a child of 20 kg varies from £54 to £493, and that foran adult varies from £90 to £822.50, depending on theseverity of the bleeding, and the purity, type, and quantityof FVIII used [17]. intensevascularization of oral tissues and iatrogenic trauma duringoral surgical Thus, financial concerns has forced theresearchers to study on ancillary therapies such as developingnew techniques or hemostasis protocols that mayprovide an opportunity to define what would be closer tooptimal replacement therapy by using lower doses.

Our experience as oral surgeons indicates that there isan increased risk of hemorrhage triggered by the procedures; therefore, oral care providersthat treat patients with bleeding disorders should be awareof all the measures for controlling bleeding and the principlesof atraumatic surgery. The goal is “key hole” surgeryand minimal interference with the attached gingivaaround the teeth and periosteum, so as to minimize postoperativebleeding [19]. Choice of anesthesia is also animportant issue. With the use of appropriate replacementtreatment regional anesthesia can be used; however, infiltrationanesthesia is safer than other methods. In addition,considering the direct relationship between emotional factorssuch as dental anxiety, fear of bleeding and increasedfibrinolysis, which can complicate postoperative healingin patients with bleeding disorders [1], dentists shouldperform various methods to alleviate patient stress priorto surgery. Patients with bleeding disorders would greatlybenefit from dental educational programs presented byoral care providers.

The present study has some limitations, including asmall patient population and groups consisting of patientswith various types of bleeding disorders. As the resultsof this study are considered to be preliminary, additionalresearch with larger patient populations and differentstudy designs is necessary to further investigate andcompare the efficacy of various local hemostatic agents inpatients with bleeding disorders undergoing oral surgery.

## CONCLUSION

Utilization of fibrin sealant or tissue adhesive, withonly preoperative administration of replacement therapyin patients with bleeding disorders undergoing oral surgicalprocedures safely provided rapid hemostasis at thesurgical site and facilitated performance of serial surgicalprocedures concurrently.

## CONFLICT OF INTEREST STATEMENT

The authors of this paper have no conflicts of interest,including specific financial interests, relationships, and/or affiliations relevant to the subject matter or materialsincluded.

## Figures and Tables

**Table 1:**
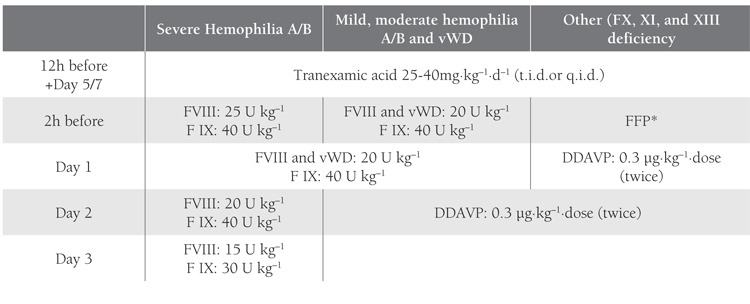
Protocol for Hemostasis

**Table 2:**
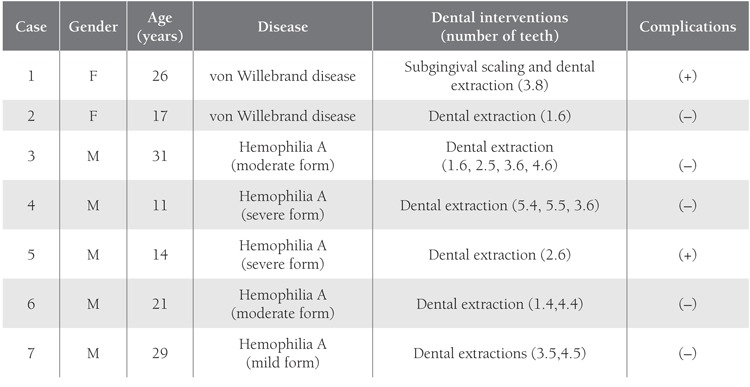
General Patient Characteristics and Dental Interventions Performed with Preoperative Replacement Therapy and LocalFibrin Sealant Application (Group 1)

**Table 3:**
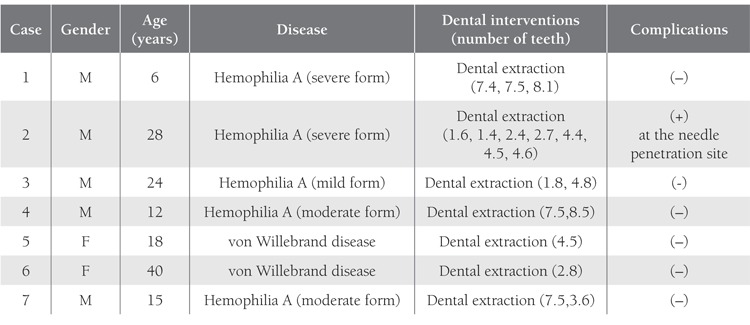
General Patient Characteristics and Dental Interventions Performed with Preoperative Replacement Therapy and Localtissue Adhesive Application in Group 2

**Table 4:**
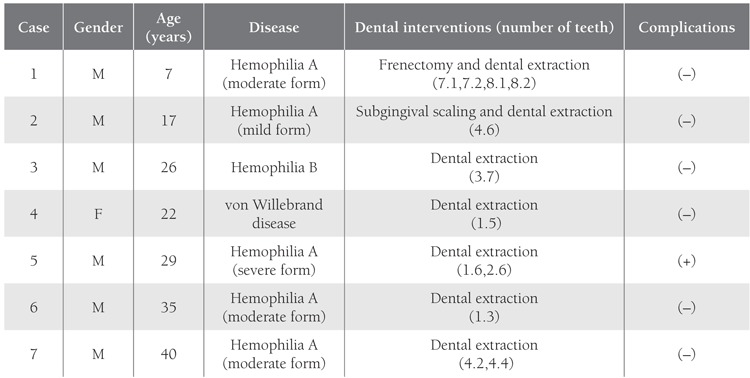
General Patient Characteristics and Dental Interventions Performed with Pre-and Postoperative Replacement Therapy inGroup 3

**Table 5:**
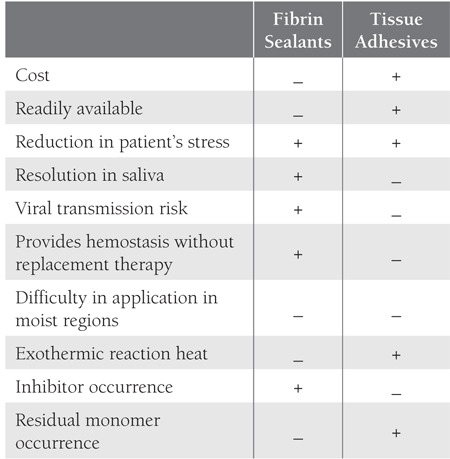
Comparison of Fibrin Sealants and Tissue Adhesives

## References

[1] Katz JO, Terezhalmy GT (1988). Dental management of the patient with haemophilia. Oral Surg Oral Med Oral Pathol.

[2] Martinowitz U, Varon D, Heim H (1998). The role of fibrin tissue adhesives in surgery of haemophilia patients.. Haemophilia.

[3] Tock B, Drohan W, Hess J, Pusatery A, Holcomb J, Machpee M (1998). Haemophilia and advanced fibrin sealant technologies. Haemophilia.

[4] Sponitz WD, Prabhu R (2005). Fibrin sealant tissue adhesivereview and update. J Long Term Eff Med Implants.

[5] Jackson MR (2001). Fibrin sealants in surgical practice: An overview. Am J Surg.

[6] Narang U (2001). Cyanoacrylate medical adhesives-a new era Colgate ORAB Soothe. N. Seal Liquid Protectant for canker sore relief. Compend Contin Educ Dent.

[7] Singer AJ, Thode HC (2004). A review of the literature on octylcyanoacrylate tissue adhesive. Am J Surg.

[8] Samuel PR, Roberts AC, Nigam A (1997). The use of Indermil (n-buthyl cyanoacrylate) in otolaryngology and neck surgery. A preliminary report on the first 33 patients. J Laryngol Otol.

[9] Piot B, Sigaud-Fiks M, Huet P, Fressinaud E, Trossaert M, Mercier J (2002). Management of dental extractions in patients with bleeding disorders. Oral Surg Oral Med Oral Pathol Oral Radiol Endod.

[10] Tseng YC, Hyon SH, Ikada Y (1990). Modification of synthesis and investigation of properties for 2 cyanoacrylates. Biomaterials.

[11] Vinters HV, Galil KA, Lundie MJ, Kauffmann JC (1985). The histotoxicity of cyanoacrylates. A selective review. Neuroradiology.

[12] Johnson WT, Leary JM (1997). Management of dental patients with bleeding disorders: Review and update. Oral Surg Oral Med Oral Pathol.

[13] Zusman SP, Lustig JP, Baston I (1992). Postextraction hemostasis in patients without reducing the dose of oral anticoagulant: The use of fibrin sealant. Quitessence Int.

[14] Suwannuraks M, Chuansumrit A, Sriudonporn N (1999). The use of fibrin glue as an operative sealant in dental extraction in bleeding disorders patients. Haemophilia.

[15] Avery BS, Ord RA (1982). The use of butyl cyanoacrylate as tissue adhesive in maxillo-facial and cranio- facial surgery. Br J Oral Surg.

[16] Kavaklı K (1999). Fibrin glue and clinical impact on haemophilia care.. Haemophilia.

[17] United Kingdom Haemophilia Centre (UKHC) Directors Organisation Executive Committee (1997). Guidelines on therapeutic products to threat haemophilia and other hereditary coagulation disorders. Haemophilia.

[18] Chandy M (2005). Management of haemophilia with minimal factor replacement in developing countries: Role of ancillary therapy. Seminars of Thrombosis and Hemostasis.

[19] Harrington B (2000). Primary dental care of patients with haemophilia. Haemophilia.

